# Routes of Motivation: Stable Psychological Dispositions Are Associated with Dynamic Changes in Cortico-Cortical Functional Connectivity

**DOI:** 10.1371/journal.pone.0098010

**Published:** 2014-06-03

**Authors:** Vanda Viola, Annalisa Tosoni, Arie W. Kruglanski, Gaspare Galati, Lucia Mannetti

**Affiliations:** 1 Department of Psychology, University of Rome “La Sapienza”, Rome, Italy; 2 Laboratory of Neuropsychology, Foundation Santa Lucia, Rome, Italy; 3 Department of Neuroscience and Imaging, University G. D’Annunzio and Institute for Advanced Biomedical Technology, Foundation G. D’Annunzio, Chieti, Italy; 4 Department of Psychology, University of Maryland, College Park, Maryland, United States of America; 5 Department of Developmental and Social Psychology, University of Rome “La Sapienza”, Rome, Italy; National Scientific and Technical Research Council (CONICET), Argentina

## Abstract

The present study provides a neurobiological framework to the theory of epistemic motivation that has been extensively studied for the last three decades in the domain of social cognition. Epistemic motivations affect the way people generate and validate hypotheses, and ultimately form and modify knowledge. Strong dispositional measures such as need for cognitive closure (NCC), the desire for a quick firm answer (any answer) to a question, show gross and stable inter-individual differences. The cognitive mechanisms and neural underpinnings of such differences, however, remain largely unexplored. Here we show that high (compared to low) levels of NCC, measured with need for cognitive closure scale, are associated with reduced online adjustment in cognitive control, as indexed by behavioral conflict adaptation. This behavioral effect is mediated by dynamic changes in cortico-cortical functional connectivity between prefrontal regions involved in conflict monitoring and implementation of cognitive control. In particular, these regions show increased functional connectivity after exposure to conflict in low but not high NCC individuals. These results demonstrate that the level of flexibility of functional cortico-cortical connections can mediate stable psychological dispositions.

## Introduction

Dispositional constructs, including personality traits and stable motivational factors, have profound and pervasive effects on cognition and behavior [1], [2]. These effects might be mediated by stable differences in brain functioning. Epistemic motivations, or motivations to acquire and construe new knowledge, appear to fulfill an essential function in the information processing enterprise. In particular, need for cognitive closure (NCC) [3]–[6] has been considered a major motivation affecting judgment formation. NCC is a cognitive-motivational content- independent construct that is individually experienced as preference for clarity, order and structure and the desire for firm and stable knowledge. Situational forces such as time pressure, environmental noise, or dullness of a task may activate a desire for closure, but NCC fundamentally reflects a dimension of stable individual differences.

The need for closure is experienced as the desire to obtain knowledge urgently and to keep it permanently. When elevated, it is known to truncate the consideration of further relevant evidence, and to promote the formation of inflexible judgments: it thus produces a “seezing” on early information and “freezing” upon the judgments it affords. In this sense, heightened need for closure fosters cognitive rigidity manifested as insensitivity to subsequent information [4], and resistance to change [7]. Functionally, the need for closure constitutes a stopping mechanism of information processing. By producing a sense of confident knowledge, it obviates the need to search for further information, thus affecting decision-making and action planning. People at the low end of the NCC continuum are characterized by a preference for variety, uncertainty, slow decision-making, flexibility of thought, and a high tolerance for ambiguity. On the other side, high NCC levels are associated with the inclination to “seize” quickly on given information, experiencing low fear of invalidity and becoming relatively impermeable to further relevant information [4], [8]–[11].

Despite at least three decades of research on this topic, it is still unclear how dispositional need for cognitive closure is related to basic differences in cognitive and neural functioning.

According to the model of political ideology as motivated social cognition [12]–[14], for example, high NCC is positively associated with political conservatism, an association that has been also supported by empirical evidence [15], [16]. According to this model, the typical tendency to justify and preserve the status quo of the conservative political ideology provides the best fit to individual’s high levels of NCC.

Behavioral research suggests that psychological differences between conservatives and liberals could be explained by differences in self-regulatory process of conflict monitoring [17]. Conflict monitoring can be described as general mechanism for the detection of situations in which an habitual response tendency is mismatched with new responses more suitable for the current situation and neuroimaging research has shown that the anterior cingulate cortex (ACC) is essential for this function [18]. On this basis, Amodio and colleagues [2] tested the hypothesis that differences between conservatives and liberals are related to differences in general neurocognitive functioning using event-related potentials. The results showed that greater liberalism was associated with stronger conflict-related anterior cingulate activity during trials requiring the inhibition of the habitual response pattern.

According to conflict monitoring theory [18]–[21] cognitive control encompasses two intertwined functions: conflict detection/monitoring and implementation of control. Specifically, the conflict monitoring theory of cognitive control asserts that increased cognitive control is recruited after the detection of a conflict, when errors are more likely [18], [22]. Conflict monitoring has been associated with activity of the ACC, which is thought to recruit regions involved in cognitive control to diminish conflict and improve subsequent performance [18], [23]–[29], while implementation of cognitive control has been associated with activity of the dorsolateral prefrontal cortex (DLPFC). When the ACC detects a cognitive conflict (either because an error has been committed or because multiple competing response options are available), it “triggers” DLPFC to implement control at the response level [28], [29]. The DLPFC minimizes conflict by sending top-down biasing signals to frontal and posterior systems that consequently reduce conflict and increase strategic focus [26], [30], [31].

In experimental psychology, the context-sensitive recruitment of cognitive control processes has often been studied using conflict tasks. In these tasks, the recruitment of cognitive control processes is measured as the amount of the “congruency effect”, representing the difference in response time (RT) between incongruent (response conflict) and congruent (no response conflict) trials. This congruency effect represents the additional time necessary to resolve increased response conflict in incongruent compared to congruent trials. For instance, in the Flanker task [32], participants are instructed to respond to a centrally presented target (e.g. letters or symbols) but have to disregard surrounding flanker stimuli. Trials in which the response indicated by the target and the one indicated by the flanker stimuli are incongruent typically produce increased RT. This congruency effect can be modulated by additional context variables implicating processes of control adjustments: conflict in the previous trial modulates the recruitment of control, leading to a reduced congruency effect following incongruent trials [33]. Because of these adjustments in the level of control from trial to trial, this “congruence sequence effect” represents a very sensible measure of flexibility in cognitive control processes.

Consistent with the conflict monitoring hypothesis, neuroimaging studies have shown that the behavioral adjustments in response to conflict detection such those observed in the congruence sequence effect are associated with a decrease of activity in the ACC and a subsequent increase of activity in the DLPFC [22], [28].

On this basis and core definitional tendencies of NCC to “seeze” on early information and “freeze” upon the judgments it affords, we predict that individual levels of NCC are correlated with flexibility, both measured as online adjustment in cognitive control and as degree of functional cortico-cortical connections between the ACC and DLPFC. In particular, we hypothesize that high levels of NCC bring, as conservatism does, to a lower flexibility in the context-sensitive recruitment of cognitive control. This reduced flexibility in subjects with high levels of NCC would produce an impairment of mechanisms for adaptation to behavioral conflict and a concurrent reduction of the functional connection between the ACC and DLPFC regions.

To test these predictions, we asked participants with different levels of NCC to perform the Flanker task to measure levels of cognitive control and behavioral adjustments (i.e. congruence effect and congruence sequence effect).

Specifically, we conducted two consecutive studies adopting an approach based on individual differences: in the first study we analyzed whether dispositional levels of NCC affected performance on a Flanker task [32]. In the second study we used functional magnetic resonance imaging (fMRI) to assess brain activity and functional connectivity during the same task.

## Study 1

The first study aimed at testing the hypothesis that individuals with high levels of need for cognitive closure, as compared to individuals with low levels of NCC, show lower adaptation to behavioral conflict. To this aim participants with low and high NCC were administered the Eriksen flanker task [32].

## Methods

### Participants

**Figure 1:**
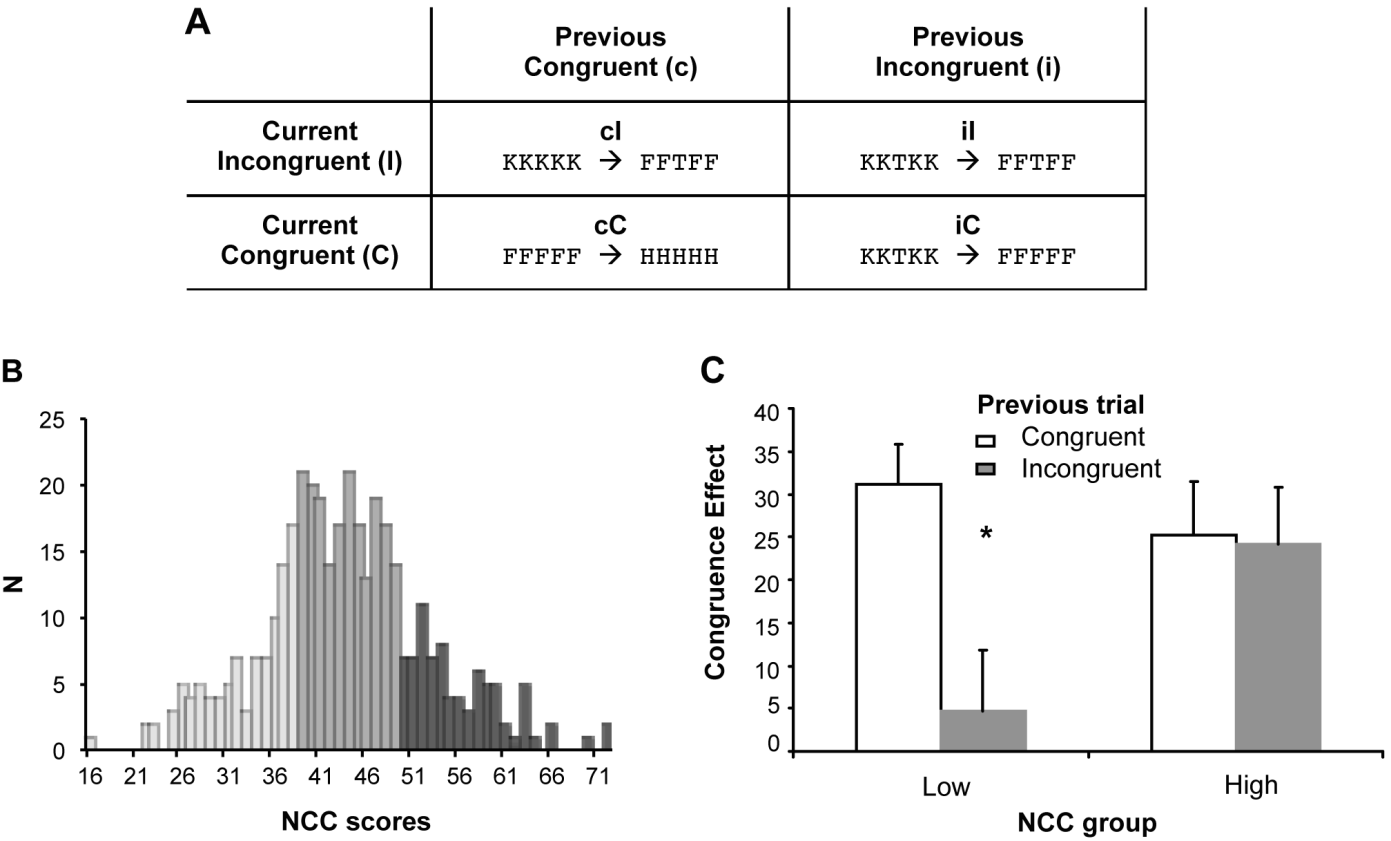
Experimental design and behavioral data. **A:** Schematic representation of the Eriksen Flanker task. The letter strings represent examples of consecutive trials. **B:** Distribution of NCC scores in 373 volunteers. The gray scale indicates the three sub-groups with low, high and medium levels of NCC: pearl gray corresponds to the first quartile (low NCC), middle grey to the second and third quartile (medium NCC), and dark grey to the fourth quartile (high-NCC). **C:** Congruence effect on current trial (RTs on Incongruent–Congruent trials) as a function of congruence in previous trial and NCC level (low and high NCC). The asterisk represents a significant reduction of the congruence effect following an incongruent trial.

Three hundred and seventy three volunteers (18–36 years old) completed a survey on the Need for Cognitive Closure Scale (NCC) [34]. Based on the scores on the NCC scale, we next divided the total sample of subjects into four quartiles and randomly selected for the behavioral study only subjects of the 4th (22 subjects with high NCC; mean NCC = 57.36, standard deviation, s.d. = 5.02) and 1st quartile (21 subjects with low NCC, mean NCC = 31.66, s.d. = 4.48) (Figure 1B). Groups were matched for age and gender (24 females, mean age = 30.07; s.d. = 3.45).

All participants gave written informed consent to participate in the study, which was approved by the Ethics Committee of the Santa Lucia Foundation (Scientific Institute for Research Hospitalization and Health Care). None of the participants had a history of neurological or psychiatric disorders.

### Need for Cognitive Closure Questionnaire

NCC was measured with a short version of the Italian Need for Cognitive Closure scale devised by Pierro & Kruglanski [34]and already used in previous studies [35]. The scale consists of 14 items, all loaded on one factor (e.g. “When I find myself facing various, potentially valid, alternatives, I decide in favor of one of them quickly and without hesitation”, “I get very upset when things around me aren’t in their place”, “Any solution to a problem is better than remaining in a state of uncertainty”. Additionally 2 filler items were included in the scale but not considered in the final score (see Appendix S1). In this study reliability of the scale measured with Cronbach’s scale was equal to 0.772. Responses to items were rated on a 6-point scale ranging from 1 (strongly disagree) to 6 (strongly agree), with higher scores indicating a greater need to attain cognitive closure. The total score, thus, varied from 14 (lowest NCC) to 84 (highest NCC).

### Experimental Task

The Eriksen Flanker task [32] has been commonly used to test the conflict monitoring theory predictions. In this paradigm, target and flanker stimuli either trigger the same response (congruent, or low conflict trials) or different responses (incongruent, or high conflict trials). Response times are typically longer on incongruent (I) than congruent (C) trials (congruence effect). However, individuals also respond more quickly and more accurately to incongruent trials that follow an incongruent trial (iI) than to incongruent trials that follow a congruent trial (cI: see also Figure 1A). This effect, known as “congruence sequential effect” [33], is generally considered an index of behavioral conflict adaptation [36]; [37] and provides “a direct window onto online adjustment in cognitive control” [56]. This effect is presumably mediated by the first incongruent trial arousing cognitive openness and vigilance and hence reducing individuals’ response time to a subsequent incongruent item.

We used a modified version of the Eriksen Flanker Task [32]. Stimuli used for targets and flankers were the letters K, F, H and T. Two letters (K and F) were mapped onto the left response key, the other letters (H and T) onto the right response key. The target was presented centrally with two flankers on each side. In half of the trials target and flankers consisted of the same letters (congruent trials, e.g. KKKKK, FFFFF, HHHHH, TTTTT) while in the other half flankers were mapped onto a different response key (incongruent trials: e.g. KKHKK, FFTFF, HHKHH, TTHTT). Stimuli occurrence was counter-balanced across incongruent and congruent trials and left and right responses. Classical conflict adaptation confounds of ‘partial repetition’ (the phenomenon by which the repetition of some, but not all, the features of an event produces worse performance than repeating all or none of the features) [38], [39] and ‘repetition priming’ (the phenomenon by which the elimination of repetitions eliminates the conflict-adaptation pattern) [36] were controlled by randomizing the trial sequence such that the proportion of repetitions and alternations was close to 50% across all trials. Letters were black strings 5.71 dva wide presented on a white background. Each trial began with the presentation of a white mask for 33 ms, followed by the five-letter string for 80 ms and by the white mask again for 33 ms. At the end of the trial, a 0.6 dva wide fixation cross was centrally presented on the screen. Subjects were required to respond to the central letter by pressing the corresponding response key as quickly and accurately as possible. No explicit performance feedback was given during the experiment. Before the behavioral or fMRI sessions, participants practiced the task until they reached 80% accuracy.

### Procedure

The experiment took place in a dimly lit room. Participants were seated in front of the computer monitor at 40 cm of distance and completed three consecutive sessions of 5 min duration each. Each session included 225 trials presented every 1.5 s. Trials subdivision across conditions were as follows: 112 congruent trials (C), of which 56 were preceded by another congruent trial (cC trials) and 56 by an incongruent trial (iC trials), and 112 incongruent trials (I), of which 56 were preceded by a congruent trial (cI trials) and 56 by an incongruent trials (iI trials) (see Figure 1A). The first trial of each session was not considered in the analysis because it could not be assigned to any of the condition sequences (e.g cI, cC, iC, iI). Subjects were instructed to respond by pressing one of two buttons on a response pad (Cedrus Co., San Pedro, CA, USA).

### Data Analysis

Behavioral data were analyzed as follows. Upper and lower limits of two standard deviations from the mean response time (RT) were set to highlight potential outliers. Based on these criteria, 5 subjects (2 belonging to high and 3 low NCC group) were discarded as outliers because of their RTs being higher than 2 standard deviations from the mean RT. Incorrect trials (congruent trials: 2,01%, s.d. 2.13; incongruent trials; 2.11%, s.d. 2.86) were also excluded from the analysis. To test whether individual levels of NCC significantly affected behavioral measures of conflict monitoring and adaptation, we conducted a series of mixed-effects analyses of variance (ANOVAs) with Congruence (congruent, incongruent) of the current and/or of the previous trial as within-subject factor(s) and NCC (low vs. high) as a between-subject factor on participants’ RTs.

## Results

First of all, we examined whether the CE, i.e., the RT increase in incongruent relative to congruent trials, was different in individuals with low vs. high levels of NCC. To this aim, we performed an ANOVA with Congruence of the current trial (congruent, incongruent) and NCC (high vs. low) as factors. Results indicated a main effect of Congruence (F_1.36_ = 63.95; p = 0.01) but not a significant effect of NCC (F_1.36_ = 0.76; p = 0.39) or a significant NCC by Congruence interaction (F_1.36_ = 0.05; p = 0.83) (Figure S1). This indicated that a significant CE was present in both high and low NCC participants.

We next tested the effect of NCC on conflict adaptation by examining the congruence sequential effect (CSE), i.e., the reduction of the congruence effect in the trial subsequent to an incongruent trial. To this aim, we first estimated the congruence effect in each subject by subtracting RTs for congruent trials from RTs for incongruent trials, separately for trials following congruent and incongruent trials, and then conducted an ANOVA with Congruence of the previous trial and NCC as factors. Results showed a significant interaction of Group by Congruence (F1.36 = 5.13; p = 0.03). As shown by Newman-Keuls post-hoc tests, the interaction was explained by the presence of a significant CSE in the low NCC group (p = 0.01) but not in the high NCC group (p = 0.60) (Figure 1C). Thus, extreme levels of NCC modulate conflict adaptation, as indexed by the CSE.

## Study 2

The second study aimed at assessing whether the modulation of the congruence sequential effect by NCC was associated with, and mediated by, different levels of functional communication between brain regions involved in conflict monitoring and implementation of cognitive control. In other words, we intended to test whether the absence of conflict adaptation effects in high NCC participants was associated with lower levels of functional communication between specific cortical regions selected from the BOLD effect of Congruence on the same participants.

To this aim, participants performed the same Flanker task as in Study 1 while fMRI activity was recorded.

## Methods

### Participants

The fMRI study involved twenty nine subjects [15 females, 18–35 years old, mean age = 28.10, s.d. = 3.56. 14 subjects with low NCC (with scores included in the I quartile: mean = 34.21, s.d. = 4.14) and 15 subjects with high NCC (with scores included in the IV quartile: mean = 53.87, s.d. = 5.49)] that were different from those participating in the behavioral experiment but selected from the same sample of volunteers that completed the survey on the Need for Cognitive Closure Scale (NCC) described above.

### Functional Magnetic Resonance Procedure

Each participant underwent six consecutive fMRI acquisition scans of 190 functional MR images, lasting approximately 6 min each. During each scan, 126 trials were presented every 2.5 s. Although a longer inter trial interval (ITI) would increase the efficiency of the event-related fMRI design (Dale et al., 1999), the congruence sequence effect is most apparent with such low ITIs. Three 25-s rest blocks, in which a fixation cross was shown, were inserted at the beginning, at the middle, and at the end of each scan. The first trial of each scan and the first trial after a rest block at the middle of each scan were excluded from the analysis. The remaining 124 trials included 62 congruent trials (31 preceded by a congruent [cC] and 31 by an incongruent [iC] trial), and 62 incongruent trials (31 preceded by a congruent [cI] and 31 by an incongruent [iI] trial). Trials were arranged in a pseudo-randomized order, which was different every two scans but fixed across subjects. As in the behavioral study, reaction times and correct responses were recorded.

Images were acquired on a 3T Siemens Allegra MR system (Siemens Medical Systems, Erlangen, Germany) operating at Foundation Santa Lucia, using a standard head coil. A control computer located outside the MR room generated stimuli by running in-house software written in Cogent 2000 (developed at FIL and ICN, UCL, London, UK) and implemented in MATLAB (The MathWorks Inc., Natick, MA, USA).

Echo-planar functional MR images (TR = 2 s, TE = 30 ms, flip angle = 70°, 64×64 image matrix, 3×3 mm in-plane resolution, 30 slices, 3 mm slice thickness with a 0.8 mm gap, sequential excitation order) were acquired in the AC–PC plane using blood-oxygenation-level-dependent imaging [57]. From the superior convexity, sampling included all the cerebral cortex. A three-dimensional high-resolution anatomical image was also acquired for each subject (Siemens MPRAGE sequence, TR = 2 s, TE = 4.38 ms, flip angle = 8°, 512×512 image matrix, 0.5×0.5 mm in-plane resolution, 176 contiguous 1 mm thick sagittal slices).

### Pre-processing and Data Analysis

Functional magnetic resonance images were pre-processed and analyzed using SPM8 (Wellcome Department of Cognitive Neurology, London, UK). Functional MR images from each subject were first spatially corrected for head movement, using a least-squares approach and six-parameter rigid body spatial transformations, and then temporally corrected for slice timing, using the middle slice acquired in time as a reference. Functional data were then spatially normalized using an automatic nonlinear stereotaxic normalization procedure (final voxel size: 2×2×2 mm) and spatially smoothed with a three-dimensional Gaussian filter (6 mm full width at half maximum). The template image for spatial normalization was based on average data provided by the Montreal Neurological Institute [40] and conforms to a standard coordinate referencing system [41].

Images were analyzed using a standard random-effects procedure. At the first stage, the time series of functional MR images obtained from each participant was analyzed separately. The effects of the experimental paradigm were estimated on a voxel-by-voxel basis, according to the general linear model (GLM) extended to allow the analysis of fMRI data as a time series. The onset of each trial constituted a neural event that was modeled using a canonical hemodynamic response function, chosen to represent the relationship between neuronal activation and blood flow changes. Separate regressors were included for each trial type (cI, iI, iC, cC), yielding separate parameter estimates for the average hemodynamic response evoked by each condition. Because incorrect responses occurred in very few trials (2% of the total trials) they were modeled as separate regressors and not further analyzed. At the second stage, linear compounds of subject-specific images of parameters estimates were entered in group analyses in which subject was treated as a random effect. The resulting statistical parametric maps (T statistics) were threshold at p<0.05, corrected for multiple comparisons using false discovery rate (FDR) [42].

**Figure 2:**
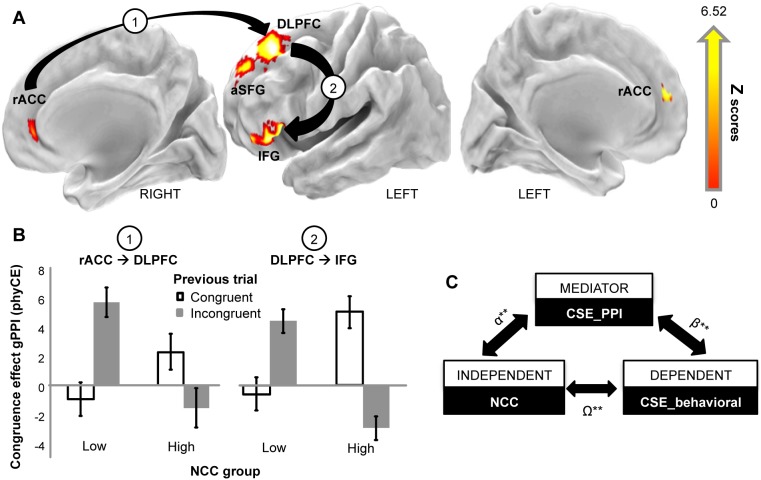
fMRI data. **A:** Brain regions showing differential BOLD responses to incongruent vs. congruent trials. Activations are rendered onto the PALS atlas. Arrows indicate the connections between rACC and DLPFC and between DLPFC and IFG, showing near-significant and significant gPPI, respectively. **B:** Congruence effect on the rACC-DLPFC and DLPFC-IFG gPPI, as a function of congruence in previous trial. Data are presented as in Figure 1C. **C:** Hypothesized mediational model linking NCC to the behavioral and gPPI CSE indices.

In order to identify patterns of brain activation associated with the congruence sequential effect (CSE) and their differences across the two groups, we first selected brain regions exhibiting higher activation during incongruent vs. congruent trials using a conventional voxel-wise random-effects analysis (Figure 2A, main effect of CE: p<0.05 FDR; spatial extent>100 mm^3^) and then tested the effect of NCC (low s. high), Congruence of the current trial, and Congruence of the previous trial on the average regional BOLD signal in each of the activation clusters resulting from this statistical map.

We next estimated the functional integration between all possible region pairs using a generalized form of psychophysiological interactions (gPPI) [43]–[45]. gPPI analysis models BOLD responses in one target brain region in terms of the interaction between a psychological process (here, the Eriksen Flanker Task trials) and the neural signal from a source region. In other terms, gPPI allows to test how specific experimental conditions modulate functional connectivity between the source and the target region. Specifically, for each subject and each pair of source and target regions, we modeled the BOLD signal in the target region as a (linear) combination of: (a) the effects of the experimental conditions (cC, cI, iC, iI), modeled through canonical hemodynamic functions (see above for details); (b) a regressor containing the BOLD time course of the source region, which modeled “intrinsic” functional connectivity between the source and the target region, i.e., the relationship between BOLD time courses in the source and target regions independently from the experimental condition; and (c) four further regressors expressing the interaction between trial-induced activation and neural signal in the source region. These interaction terms are called psycho-physiological interactions, because they model how variations in neural signals in the source region (physiological effect) modulate the effect of an experimental condition (psychological effect) on neural activity in the target region. In other words, psycho-physiological interactions model the increase/decrease of functional connectivity between the source and the target region during a given experimental condition. Technically, gPPI regressors were built as described in Gitelman et al. [44] and assembled as described in McLaren et al. [43]. The resulting parameter estimates (gPPI estimates) are expressed as percent signal changes in BOLD signal in the target region as a function of percent signal change in the seed region.

At the group level, we tested whether gPPI estimates, i.e., trial-contingent modulations of functional connectivity, were modulated by the congruence of the current and/or of the previous trial and by individual NCC levels. gPPI estimates were analyzed as for behavioral data, through a series of mixed-effects ANOVAs with NCC and Congruence of the current and/or of the previous trial as factors.

A further analysis tested the hypothesis that the causative effect of dispositional NCC levels on behavior was mediated by functional connectivity. To this aim, we used a basic mediation analysis [46]. A bootstrapping method (n = 1000 bootstrap resamples) was used to test the reliability of the indirect effects in this possible model of mediation.

## Results

### Behavioral Results

RT data collected in this study were analyzed as for study 1. Results replicated the effects shown in the behavioral study, despite the smaller sample. We indeed observed a significant effect of Congruence of the current trial (F_1.27_ = 33.04; p = 0.0001), with no significant effect of NCC (F_1.27_<0.01; p = 0.99) nor a significant NCC by Congruence interaction (F_1,27_ = 2.33; p = 0.13). We also replicated a significant interaction between NCC and Congruence of the previous trial on the CE (F_1.27_ = 5.73; p = 0.02) (Figure S2), which confirmed that NCC moderates the congruence sequential effect: the effect was present in low but not in high NCC individuals (Newman Keuls post hoc tests: low NCC, p = 0.01; high NCC, p = 0.60).

### Brain Activation

The group-level whole-brain map of the main effect of Congruence identified five frontal regions in which the BOLD signal was significantly higher on incongruent than congruent current trials (Figure 2A; Table S1): bilateral rostral anterior cingulate cortex (rACC); left dorsolateral prefrontal cortex (DLPFC); left inferior frontal gyrus (IFG); left anterior superior frontal gyrus (aSFG).

The left rACC and the left aSFG exhibited significantly higher activity in high than in low NCC individuals (main effect of Group: left rACC, F1.27 = 5.07; p = 0.03; left aSFG, F1,27 = 5.57; p = 0.03). Furthermore, all five regions showed a significant increase of activity when the previous trial was incongruent (main effect of Congruence of the previous trial, all p<0.05). This results indicate that the same regions responsive to the congruence of both current and previous trials are differently activated as a function of NCC levels (Figure S3). However, in none of the regions we found evidence for an interaction between Group and Congruence of either the current or the previous trial.

### Functional Connectivity

Starting from the five regions described above, we first analyzed “intrinsic” functional connectivity at rest between each region pair as a function of NCC level. The results indicated that these regions showed a positive functional connectivity at rest (e.g., DLPFC-IFG: 0.56, s.d. 0.79; one-sample t-test vs. 0: T1,27 = 3.84, p = 0.002 Bonferroni Corrected; ACC-DLPFC: 0.69, s.d. 0.85; T1,27 = 4.38, p = 0.002 Bonferroni Corrected). However, no group differences were found in these resting-state functional connectivity data, hence suggesting that dispositional levels of NCC may affect functional connectivity only during the experimental task.

To test this prediction we used gPPI analysis providing estimates of functional connectivity modulations in each of the four types of experimental trials (cC, cI, iC, iI) independently and separately for the two NCC groups. In general, we found that functional connectivity between all possible pairs of the five examined regions increased during task execution relative to rest in both groups (e.g., DLPFC-IFG: 2.02, s.d. 3.26; T1.27 = 3.34, p = 0.004 Bonferroni Corrected). However, to test whether functional connectivity was modulated by individual level of NCC and behavioral measures of conflict monitoring and conflict adaptation, we used these gPPI estimates as dependent variables of a series of ANOVAs with Group (low, high NCC) and Congruence (congruent, incongruent) of the current and/or of the previous trial as factors. In particular, to investigate a possible physiological counterpart of the CE (phyCE), defined as the increase of neural communication between two regions during incongruent vs. congruent trials, we first analyzed gPPI estimates as a function of Group and Congruence of the current trial. Note that a positive phyCE indicates an increase of functional connectivity during incongruent vs. congruent trials, while a negative value indicates the opposite pattern. We found no effect of the current trial type (C, I), neither a Group by Congruence interaction (all p>0.05).

We next tested whether functional connectivity was modulated by the congruence of the previous trial by investigating a possible physiological equivalent of the CSE (phyCSE), defined as an increase of the phyCE after an incongruent trial. To this aim, we conducted an ANOVA with Group and Congruence of the previous trial as factors on the phyCE of all possible region pairs. The results indicated that the phyCE between the DLPFC (seed region) and the IFG (target region) exhibited a significant Group by Congruence of the previous trial interaction (F1,27 = 6.22; p = 0.038 Bonferroni Corrected: Figure 2B, right panel), with post-hoc tests (Duncan) indicating a significant increase of the phyCE after an incongruent trial in the low (p = 0.05) but not in the high NCC group (p = 0.20). A similar pattern was evident for the connectivity between the right ACC (seed region) and the DLPFC (target region), although the interaction was not significant (F1,27 = 3.87; p = 0.118 Bonferroni Corrected: Figure 2B, left panel). These results indicate that only the DLPFC-IFG connection was differently modulated in the two groups by the interaction between the congruence of the current and the previous trial. The increase in functional connectivity observed during incongruent trials was positively modulated by the incongruence of the previous trial only in individuals with low NCC. In other words, the phyCSE [(iI – cI) - (iC - cC)] was higher in individuals with low than high NCC.

We lastly examined whether the phyCSE was linearly associated with the behavioral CSE (behCSE) and whether it could directly mediate the causative effect of dispositional NCC levels on behavioral measures of conflict adaptation. To this aim, we first tested the existence of a linear relationship between the behCSE estimated in each subject from behavioral data and the phyCSE estimated from PPIg values. The results indicated a significant correlation for the DLPFC-IFG but not for the ACC-DLPFC connectivity (DPLFC-IFG: r = −0.55, p = 0.004, Bonferroni-corrected; ACC-DLPFC: r = −0.24, p = 0.388, Bonferroni-corrected). A negative correlation indicates that an increase of communication between the DLPFC and the IFG after an incongruent trial was associated with a decrease of the behavioral congruence effect after an incongruent trial.

We next tested whether the influence of NCC levels (independent variable) on the behavioral CSE (dependent variable) was mediated by the connectivity between the DLPFC and the IFG, as indexed by the phyCSE (mediator), using a basic mediation analysis. This analysis showed (Figure 2C) that the indirect effect of NCC levels on the behavioral CSE index through the DLPFC-IFG phyCSE as a mediator was statistically significant (CI [−12,667, −0.676], p = 0.01). Importantly, a significant correlation was observed between the phyCSE estimated from the DLPFC-IFG connection and NCC scores (r = 0.43, p = 0.04, Bonferroni-corrected), which ensured the validity of the mediation analysis [47].

## Discussion

The goal of the present studies was to elucidate the neurocognitive mechanism underlying adaptive adjustment of cognitive control. We specifically tested whether a chronic epistemic motivation – need for cognitive closure - modulated the level of flexibility during online adjustment in cognitive control and whether this effect was mediated by specific patterns of functional interactions between different brain regions.

Results of the two independent experiments, in which performance on a Eriksen Flanker task was used as an index of behavioral conflict adaptation [19], [23], [24], [26]–[29,28], showed that chronic NCC has a significant impact on conflict-induced behavioral adjustments as measured by congruence sequential effect. In particular, we found that NCC was inversely related to participants’ tendency to respond more quickly to incongruent trials that follow an incongruent trial than to incongruent trial that follow a congruent trial. These results indicated that individuals with high NCC show a significantly lower level of cognitive adaptation then individual with low NCC consistently with their typical tendencies to “freeze” on prior notions and to be considerably slow and close-minded when considering alternative possibilities.

It is important to note that individual differences in cognitive control and self-regulation may be also related to other factors in addition to NCC. For example, as recently shown by Amodio and colleagues [2] differences in cognitive adaptation are highly correlated with political orientation. Unfortunately, however, only NCC levels were measured in the current study so it is not possible to determine whether the relation of NCC with flexibility levels during online adjustment in cognitive control is explained or mediated by other personality factors. Based on the association between NCC levels and conservatism [15], however, we suggest that such factors may be highly correlated with cognitive closure such that the typical tendency of high NCC individuals to preserve the status quo and to “freeze” on prior notions would bring, on one hand, to have a conservatism political ideology and, on the other, to exhibit a lower cognitive flexibility.

Results of the fMRI experiment showed that the behavioral difference in cognitive adaptation between the two NCC groups was mediated by the degree of functional integration in the DLPFC- IFG pathway, which has been largely associated with top-down inhibitory control [58], hence suggesting that low NCC participants, characterized by high fear of invalidity, protract and intensify functional connectivity in the control pathway (DLPFC-IFG) when presented with sequences of two incongruent trials, while this is not true for individual with high NCC. Regarding the individual differences in DLPFC response, also Hester et al. [50] showed that individual differences in the activity of this region predicted inhibitory performance.

Notably, the IFG is a core region of the ventral attention network, which is thought to control attentional reorienting to unexpected but behaviorally relevant stimuli [49]. Specifically, the IFG may have an alarm function as a part of its critical role in switching between internally and externally oriented control systems in response to salient stimuli [51].

Of interest, these differences in functional connectivity between NCC groups were not present during rest, indicating they are not due to intrinsic differences in connectivity patterns between high and low NCC participants but rather to contextual connectivity modulations associated with specific phases of the task.

Our results are also consistent with the findings of Kouneiher et al. [52] of a strong interplay between the IFG and dorsal frontal areas for the implementation a reactive immediate action in response to concomitant input signals. This is particularly relevant because in our data the incongruence was at the response level.

Moreover Egner [53] clarified the pivotal role of the IFG in cognitive control by showing that subjects displaying greater conflict-driven activity in the IFG exhibited a higher behavioral adaptation following conflicting stimuli**.**

Importantly, our data have highlighted a more specific role of NCC in conflict adaptation than in conflict detection. If we compare the typical reorienting function of the IFG with the typical conflict detection role of the ACC, we can speculate that the DLPFC-IFG connection provides a better accounts for the individual differences in cognitive adaptation than the ACC-DLPFC connection. This could explain why our results support a key role in cognitive closure of the connection between the DLPFC and the IFG regions and not of the connection between the ACC and DLPFC regions presented in the introduction.

The present study provides the first neurobiological framework to the theory of epistemic motivation that has been extensively studied for the last three decades in social psychology. These results are of twofold interest: (1) they extend the theory of need for closure to the brain level phenomena involved in knowledge formation; (2) they link a specific individual difference dimension to modulations in brain functional communication between specific brain regions associated with conflict monitoring.

Understanding the patterns of brain activation involved in closed and open mindedness determined by variations in the need for cognitive closure could furnish insights into the plethora of cognitive and social phenomena that this need underlies. Because this motivation determines judgment and decision-making processes in several domains of human activity (including individual, interpersonal, group, intergroup and cultural level phenomena) and because it is known to vary across individuals’ groups and cultures, a window into the brain level substrates could make important contributions to the psychological science of human behavior. The study of how individual differences in epistemic motivation are related to patterns of brain activity and brain connectivity surely requires further investigation. For example, it might be worth assessing whether the results obtained on chronic differences in NCC can be replicated – both at behavioral and brain level - with induced NCC, and whether the effect of NCC on flexibility of cognitive control also applies to mechanisms of decision-making, such for example those involving moral dilemmas [54], [55], or those involving political choices [2].

## Supporting Information

Figure S1 **Congruence Effect on current trial.** The bar plot shows response times (RT) as a function of NCC group and of the congruence of the current trial (C;I) during the Study 1.
(TIFF)

Figure S2 **Congruence Effect on current and previous trial.** The bar plot shows the congruence effect (CE: RTs on Incongruent – Congruent current trials), as a function of NCC group and of the congruence of the previous trial during the Study 2.
(TIFF)

Figure S3 **fMRI data on congruence effect on current and previous trial.** The bar plots show the BOLD response in regions in which the BOLD signal was significantly higher on incongruent then congruent trials as a function of two NCC groups and congruence of the previous trial. From the left to the right the regions are: the dorsolateral prefrontal cortex (DLPFC-lh), the anterior superior frontal gyrus (aSFG-lh), the inferior frontal gyrus (IFG-lh) and the bilateral anterior cingulate cortex (r-ACC-lh; r-ACC-rh).
(TIFF)

Table S1 **Regions exhibiting a main effect of Congruence on current trial.**
(DOC)

Appendix S1 **Need for Cognitive Closure Scale.**
(DOC)
