# Supplementary figures and images for: Routes of Motivation: Stable Psychological Dispositions Are Associated with Dynamic Changes in Cortico-Cortical Functional Connectivity
Source: PLoS One. 2014 Jun 3;9(6):e98010. doi: 10.1371/journal.pone.0098010 (PMC4043525; doi:10.1371/journal.pone.0098010)

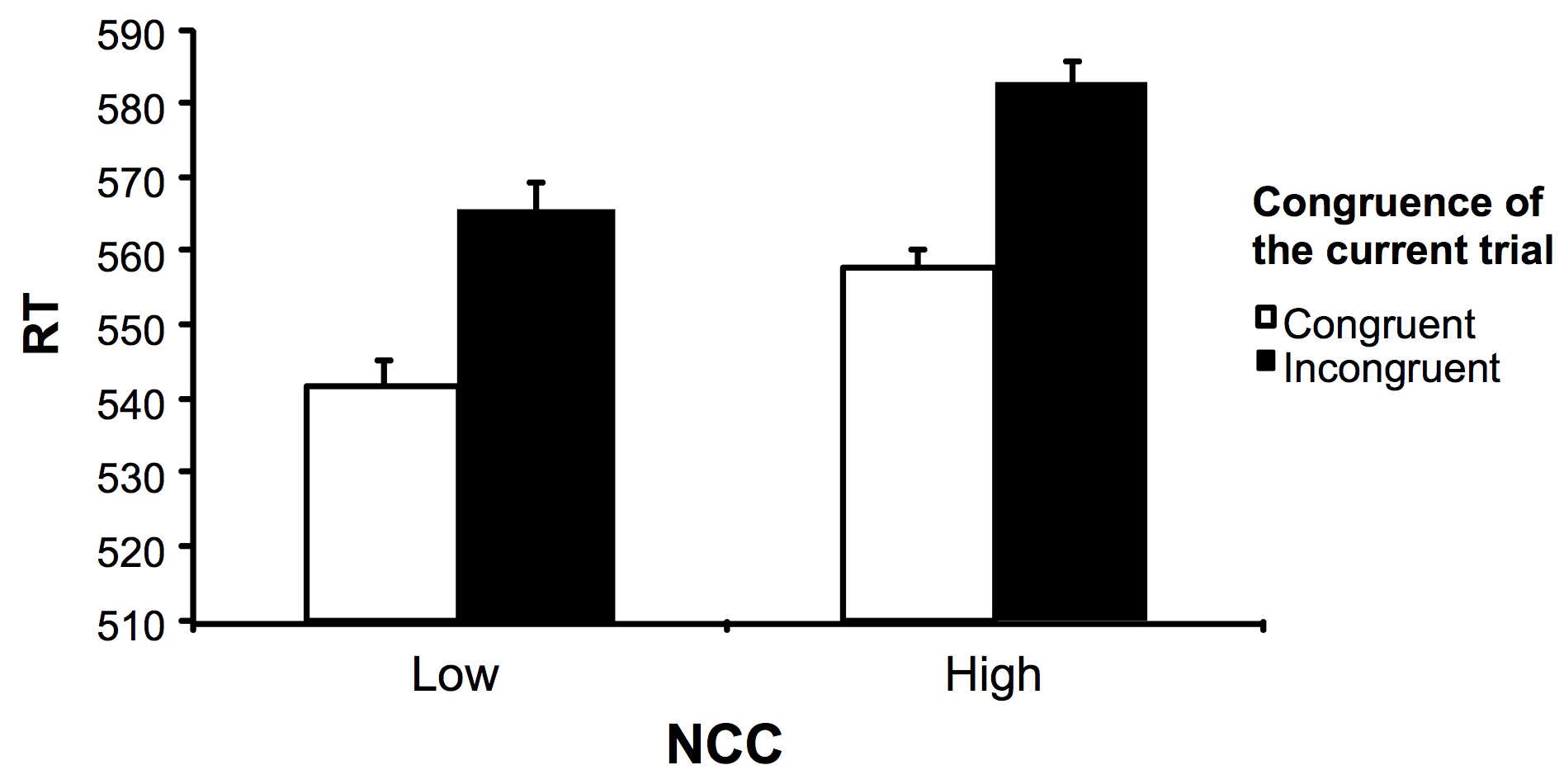

Supplement: Figure S1 — Congruence Effect on current trial. The bar plot shows response times (RT) as a function of NCC group and of the congruence of the current trial (C;I) during the Study 1. (TIFF) [file pone.0098010.s001.tiff]

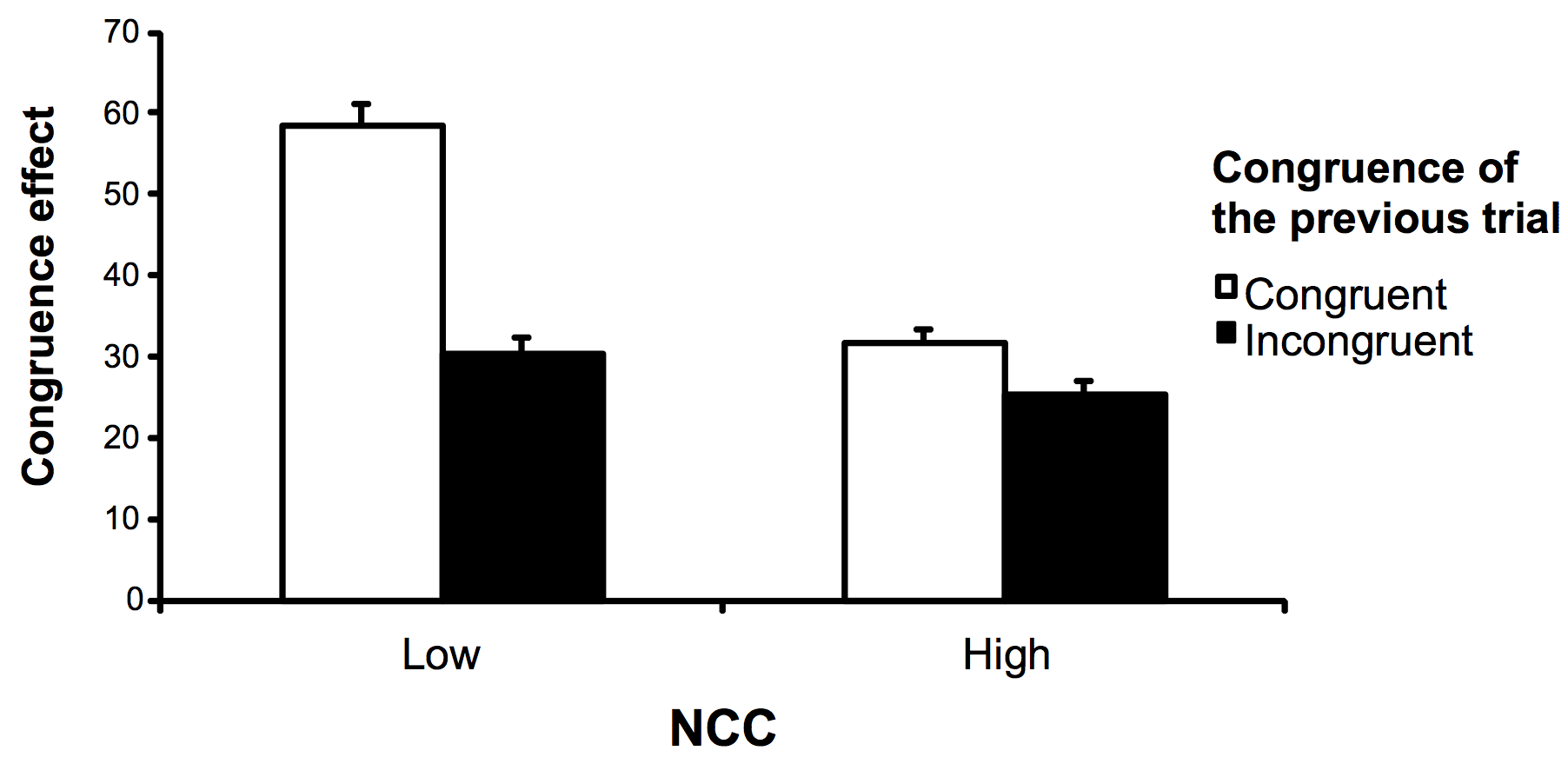

Supplement: Figure S2 — Congruence Effect on current and previous trial. The bar plot shows the congruence effect (CE: RTs on Incongruent – Congruent current trials), as a function of NCC group and of the congruence of the previous trial during the Study 2. (TIFF) [file pone.0098010.s002.tiff]

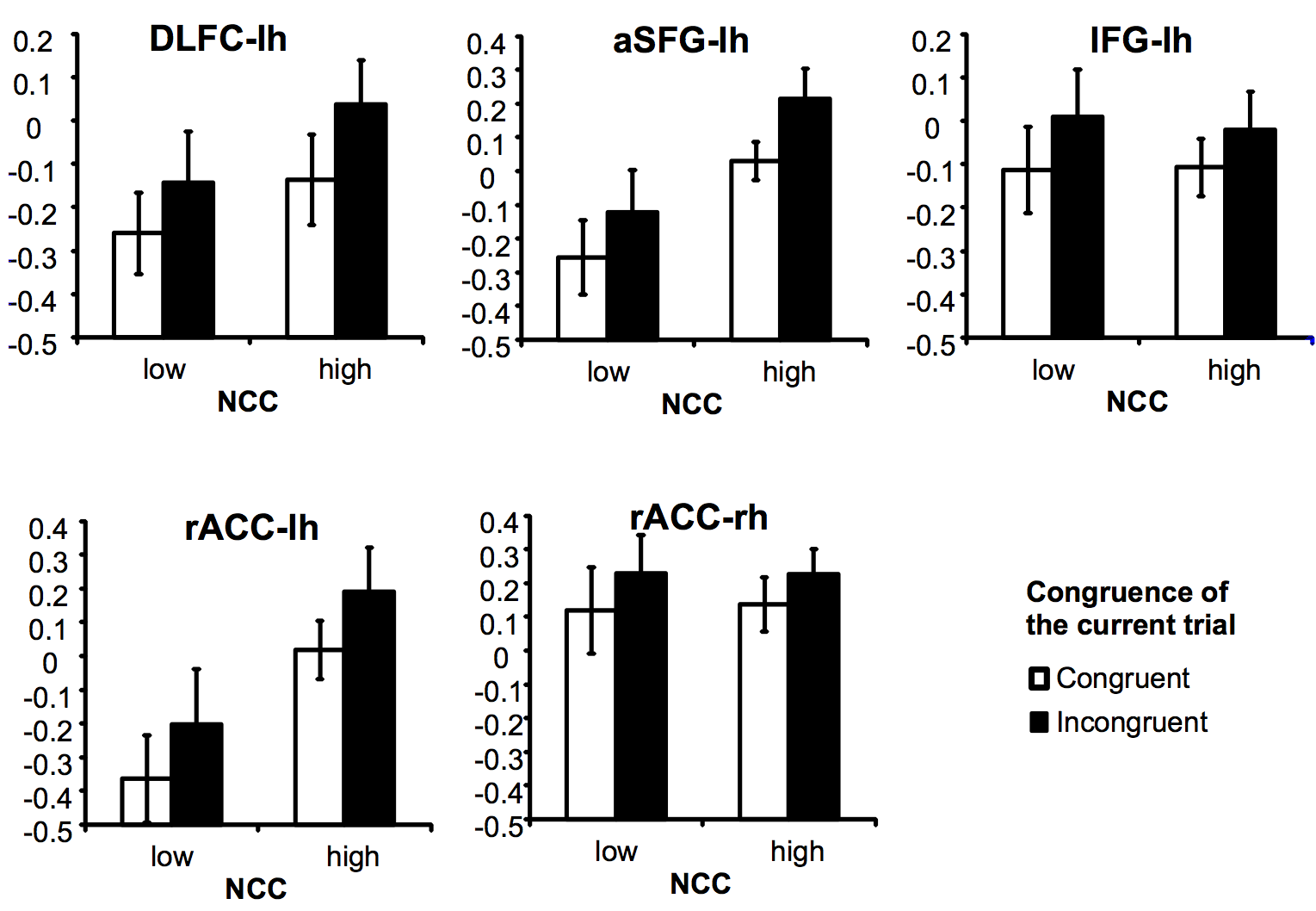

Supplement: Figure S3 — fMRI data on congruence effect on current and previous trial. The bar plots show the BOLD response in regions in which the BOLD signal was significantly higher on incongruent then congruent trials as a function of two NCC groups and congruence of the previous trial. From the left to the right the regions are: the dorsolateral prefrontal cortex (DLPFC-lh), the anterior superior frontal gyrus (aSFG-lh), the inferior frontal gyrus (IFG-lh) and the bilateral anterior cingulate cortex (r-ACC-lh; r-ACC-rh). (TIFF) [file pone.0098010.s003.tiff]
