# Supplementary material for: Routes of Motivation: Stable Psychological Dispositions Are Associated with Dynamic Changes in Cortico-Cortical Functional Connectivity
Source: PLoS One. 2014 Jun 3;9(6):e98010. doi: 10.1371/journal.pone.0098010 (PMC4043525; doi:10.1371/journal.pone.0098010)
Supplement: Table S1 — Regions exhibiting a main effect of Congruence on current trial. (DOC) [file pone.0098010.s004.doc]

| N | Region | Hemisphere | BA | Cluster size (mm^3^) | MNI coordinates  x y z | | | T_1,28_ |
| --- | --- | --- | --- | --- | --- | --- | --- | --- |
| 1 | DLPFC | L | 9 | 4131 | -21 | 35 | 49 | 5.81 |
| 2 | IFG | L | 45 | 3402 | -48 | 35 | -2 | 4.78 |
| 3 | aSFG | R | 9 | 3753 | -15 | 53 | 28 | 5.73 |
| 4 | rACC | L | 10 | 918 | -9 | 56 | 16 | 4.45 |
| 5 | rACC | R | 11 | 2673 | 18 | 41 | -5 | 5.27 |

^a^ BA = Brodmann area; L = left; R = right; MNI = Montreal Neurological Institute.

^b^ Significant at FDR-corrected p<0.05
