# Supplementary material for: Routes of Motivation: Stable Psychological Dispositions Are Associated with Dynamic Changes in Cortico-Cortical Functional Connectivity
Source: PLoS One. 2014 Jun 3;9(6):e98010. doi: 10.1371/journal.pone.0098010 (PMC4043525; doi:10.1371/journal.pone.0098010)
Supplement: Appendix S1 — Need for Cognitive Closure Scale. (DOC) [file pone.0098010.s005.doc]

Need for Cognitive Closure Scale

ATTITUDES, BELIEFS, AND EXPERIENCES SURVEY

Read each of the following statements and decide how much you would agree with each according to your attitudes, beliefs and experiences. Please respond according to the following scale, using only one number for each statement.

|  |  | 1 = Strongly disagree |  |  | 4 = Slightly agree |  |  |
| --- | --- | --- | --- | --- | --- | --- | --- |
|  |  | 2 = Moderately disagree |  |  | 5 = Moderately agree |  |  |
|  |  | 3 = Slightly disagree |  |  | 6 = Strongly agree |  |  |

| 1 | In case of uncertainty, I prefer to make an immediate decision, whatever it may be. | 1 2 3 4 5 6 |
| --- | --- | --- |
| 2 | When I find myself facing various, potentially valid, alternatives, I decide in favor of one of them quickly and without hesitation. | 1 2 3 4 5 6 |
| 3 | I have never been late for work or for an appointment. | 1 2 3 4 5 6 |
| 4 | I prefer to decide on the first available solution rather than to ponder at length what decision I should make. | 1 2 3 4 5 6 |
| 5 | I get very upset when things around me aren’t in their place. | 1 2 3 4 5 6 |
| 6 | Generally, I avoid participating in discussions on ambiguous and controversial problems. | 1 2 3 4 5 6 |
| 7 | When I need to confront a problem, I do not think about it too much and I decide without hesitation | 1 2 3 4 5 6 |
| 8 | When I need to solve a problem, I generally do not waste time in considering diverse points of view about it. | 1 2 3 4 5 6 |
| 9 | I prefer to be with people who have the same ideas and tastes as myself. | 1 2 3 4 5 6 |
| 10 | Generally, I do not search for alternative solutions to problems for which I already have a solution available. | 1 2 3 4 5 6 |
| 11 | I feel uncomfortable when I do not manage to give a quick response to problems that I face. | 1 2 3 4 5 6 |
| 12 | I have never hurt another person’s feelings | 1 2 3 4 5 6 |
| 13 | Any solution to a problem is better than remaining in a state of uncertainty. | 1 2 3 4 5 6 |
| 14 | I prefer activities where it is always clear what is to be done and how it need to be done. | 1 2 3 4 5 6 |
| 15 | After having found a solution to a problem I believe that it is a useless waste of time to take into account diverse possible solutions. | 1 2 3 4 5 6 |
| 16 | I prefer things to which I am used to those I do not know, and cannot predict. | 1 2 3 4 5 6 |
